# Knowledge, awareness, and attitudes of medical professionals toward the periodontal–systemic disease relationship: a cross-sectional study

**DOI:** 10.3389/froh.2026.1931494

**Published:** 2026-10-05

**Authors:** Narayane Ramkumar, Hanumanth Sankar, Premalatha T

**Affiliations:** 1 Department of Periodontology, Indira Gandhi Institute of Dental Sciences, Sri Balaji Vidyapeeth (Deemed to be University), Puducherry, India; 2 Department of Orthodontics, Vinayaka Mission's Sankarachariyar Dental College, Vinayaka Mission's Research Foundation (Deemed to be University), Salem, Tamil Nadu, India; 3 Vinayaka Mission's Research Foundation (Deemed to be University), Salem, Tamil Nadu, India

**Keywords:** cross-sectional study, medical professionals, oral–systemic relationship, periodontal disease, systemic diseases

## Abstract

**Background:**

Periodontal disease is a chronic inflammatory condition that has been increasingly associated with systemic diseases such as diabetes mellitus, cardiovascular disorders, respiratory diseases, and adverse pregnancy outcomes. Medical professionals play a critical role in early identification and referral; however, awareness of periodontal–systemic interactions remains variable.

**Aim:**

To assess the knowledge, awareness, and attitudes of medical professionals regarding the relationship between periodontal disease and systemic health.

**Materials and methods:**

A cross-sectional questionnaire-based study was conducted among 380 medical professionals across India, including interns, postgraduate students, practitioners, and faculty. A structured, pre-tested questionnaire was distributed electronically using Google Forms. Responses were scored using a Likert scale, and composite awareness scores were calculated. Data were analyzed using SPSS version 21. Descriptive statistics were used to summarize the data, associations between categorical variables were assessed using the chi-square test, and differences in mean awareness scores among groups were evaluated using one-way ANOVA followed by Tukey's *post-hoc* test. A *p*-value < 0.05 was considered statistically significant.

**Results:**

Although 90.5% of participants reported referring patients to dentists, only 1.1% did so based on knowledge of periodontal–systemic associations. Awareness of clinical symptoms was high (86.6%), with gingival bleeding being the most commonly identified sign (33.4%). ANOVA revealed significant differences in awareness scores among professional groups (*p* < 0.05). Government practitioners demonstrated significantly higher awareness than interns regarding diabetes mellitus and adverse pregnancy outcomes, while medical faculty demonstrated significantly higher awareness than interns regarding PCOS.

**Conclusion:**

A significant knowledge gap exists among medical professionals regarding periodontal–systemic relationships. Integrating oral health education into medical curricula and promoting interdisciplinary collaboration may improve awareness, referral practices, and patient care outcomes.

## Introduction

Oral health is a fundamental component of overall health, which plays a crucial role in maintaining both physical and psychological quality of life (1). The oral cavity serves as a gateway capable of influencing systemic health by harboring complex microbial ecosystems (2). Periodontal disease is one of the most prevalent chronic inflammatory multifactorial conditions, which affects the supporting structures of teeth, including gingiva, periodontal ligament, and alveolar bone (3). This condition arises as a result of interaction between pathogenic microbial films and host immune responses, resulting in progressive tissue destruction, tooth mobility, and eventual tooth loss (3).

Dating back to 1891, the concept of linking oral health and systemic disease emerged when Walter Miller proposed the focal infection theory, which suggested that oral microbes could disseminate to distant body sites and contribute to systemic disease processes (4). In 1996, Offenbacher strengthened this approach by using the term “periodontal medicine” to emphasize the connection between periodontal illnesses and systemic conditions (5). Since then, there has been growing evidence that demonstrates periodontal disease is not only a localized oral condition but also a potential factor for systemic health disorders (6).

Previous studies have demonstrated the correlations between periodontal disease and systemic conditions such as cardiovascular diseases, diabetes mellitus, respiratory diseases, unfavorable pregnancy outcomes, and rheumatoid arthritis (6–11). Through various mechanisms like bacteremia, dissemination of inflammatory mediators, and immune responses triggered by periodontal pathogens, chronic periodontal inflammation contributes to systemic inflammatory burden. These mechanisms may result in endothelial dysfunction, atherosclerosis, and metabolic dysregulation (12, 13).

Only a small number of studies have reported the incidence of vascular diseases among individuals with periodontal disease (14). Similarly, periodontal disease has been shown to affect the glycemic management in individuals with diabetes mellitus (7). Taylor et al. reported that severe periodontitis may be a risk factor for poor glycemic control and that managing periodontal disease may contribute to improved diabetes outcomes. A bidirectional association between these two conditions further highlights their clinical significance (7).

Periodontal disease has also been associated with adverse pregnancy outcomes. Offenbacher et al. reported that periodontal infection serves as a potential risk for preterm low birth weight (8). Experimental studies done by Collins et al. showed that infection with Porphyromonas gingivalis may result in reduction of fetal weight due to inflammatory mediator responses (15). These results suggest that periodontal health may influence maternal and fetal outcomes.

Respiratory illness has been associated with periodontal infections. Scannapieco et al. reported that oral pathogens like Pseudomonas, Staphylococcus aureus, and members of the Enterobacteriaceae family may inhabit dental plaque and be aspirated into the respiratory tract, particularly in immunocompromised individuals, contributing to respiratory infections (9). Moreover, periodontal pathogens have been associated with conditions like rheumatoid arthritis due to their ability to trigger systemic immune responses (10).

The biological mechanisms underlying these associations involve chronic low-grade systemic inflammation, which plays a central role in the pathogenesis of many systemic diseases (13). Periodontal inflammation has been shown to exacerbate insulin resistance, while poorly controlled diabetes can accelerate periodontal destruction, illustrating a bidirectional relationship (7). Similarly, inflammatory mediators produced during periodontal infection may influence the fetal–placental unit, contributing to adverse pregnancy outcomes, such as preterm birth and low birth weight, which are critical concerns for maternal and fetal health (8, 15).

Early disease detection or diagnosis by medical professionals plays an important role, as they serve as the first point of contact in the healthcare system. For comprehensive patient care, they must recognize oral manifestations of systemic diseases and understand the implications of periodontal health. However, prior research has reported insufficient awareness among medical practitioners regarding the interaction between periodontal and systemic diseases, which may result in missed opportunities for early diagnosis and timely referral (16).

It is imperative to assess the knowledge, awareness, and attitudes of medical professionals on periodontal–systemic relationships. Understanding these may help address the gaps in knowledge and be useful in directing the development of targeted educational interventions. Therefore, the present study was conducted to assess the knowledge of periodontal medicine among medical interns, postgraduate students, practitioners, and faculty across India.

## Materials and methods

### Study setting and design

This cross-sectional, questionnaire-based study involving human participants was conducted among medical professionals across India after approval from the Institutional Ethics Committee of Indira Gandhi Institute of Dental Sciences (IGIDS), Sri Balaji Vidyapeeth, Puducherry (IEC Approval Code: IGIDSIEC2021NRP49UGNDPAI). Before accessing the questionnaire, participants were informed about the study objectives, voluntary participation, anonymity, and confidentiality. Electronic informed consent was obtained through the Google Forms platform before participants proceeded to the questionnaire. The study was conducted between June 2022 and December 2022 in diverse healthcare and academic settings across the country, with participants recruited through professional networks and institutional channels.

### Inclusion and exclusion criteria

The study population comprised medical interns, postgraduate students, private practitioners, government practitioners, and medical faculty members actively engaged in clinical practice or medical training. Participants were recruited using a convenience sampling method. Inclusion criteria included individuals currently practicing medicine or undergoing medical education in India and willing to participate in the study. Those who did not provide consent or submitted incomplete responses were excluded.

A pragmatic convenience-sampling approach was used. No formal *a priori* sample-size calculation was performed. Instead, all eligible and complete responses received during the predefined recruitment period (June-December 2022) were included, yielding a final analytical sample of 380 participants. Because the questionnaire was disseminated through individual contacts, WhatsApp groups, academic networks, and group-based electronic platforms, the total number of individuals who received or viewed the invitation could not be reliably enumerated; therefore, a conventional response rate could not be calculated.

### Data collection

Data were collected using a structured and pre-tested questionnaire developed to assess knowledge, awareness, and attitude regarding periodontal disease and its systemic associations. The questionnaire comprised three sections: demographic and professional characteristics; referral practices and recognition of periodontal disease symptoms; and Likert-scale items assessing awareness of periodontal–systemic relationships. The questionnaire was pre-tested before electronic administration to ensure clarity and appropriateness. Reliability analysis demonstrated satisfactory internal consistency, with a Cronbach's alpha coefficient of 0.836 for the 13 knowledge and awareness items.

Responses to the knowledge/awareness items were coded on a five-point Likert scale from 1 to 5, according to the response options in the questionnaire. Items within each periodontal-systemic domain were assigned equal weight. For each domain, the arithmetic mean of the corresponding item scores was calculated and analyzed as a continuous composite score; higher scores indicated greater awareness. No additional categorical cut-offs were applied. The questionnaire was administered electronically through Google Forms and distributed via individual contacts, WhatsApp groups, and academic networks. Responses were collected anonymously to maintain confidentiality and minimize response bias.

Responses collected through Google Forms were exported, cleaned, coded, and analyzed in SPSS version 21 (IBM Corp., Chicago, IL, USA). Incomplete questionnaires were excluded, and only complete responses were included in the final analysis. The distribution of continuous/composite awareness scores was assessed for normality using the Shapiro–Wilk test. Descriptive statistics summarized participant characteristics and questionnaire responses. Categorical variables were expressed as frequencies and percentages and evaluated using the chi-square test. Mean awareness scores across the five professional groups were compared using one-way ANOVA, followed by Tukey's *post-hoc* test when the overall test was statistically significant. A *p*-value <0.05 was considered statistically significant.

## Results

A total of 380 medical professionals participated in the study, including interns, postgraduate students, private practitioners, government practitioners, and medical faculty. Females constituted 57.9% of the sample, and interns represented the largest proportion (36.6%), followed by postgraduates (19.2%), private practitioners (16.3%), government practitioners (16.1%), and medical faculty (11.8%).

Table 1 shows the distribution of participants by gender and professional category; the same distribution is visualized in the redesigned Figure 1.

**Figure 1:**
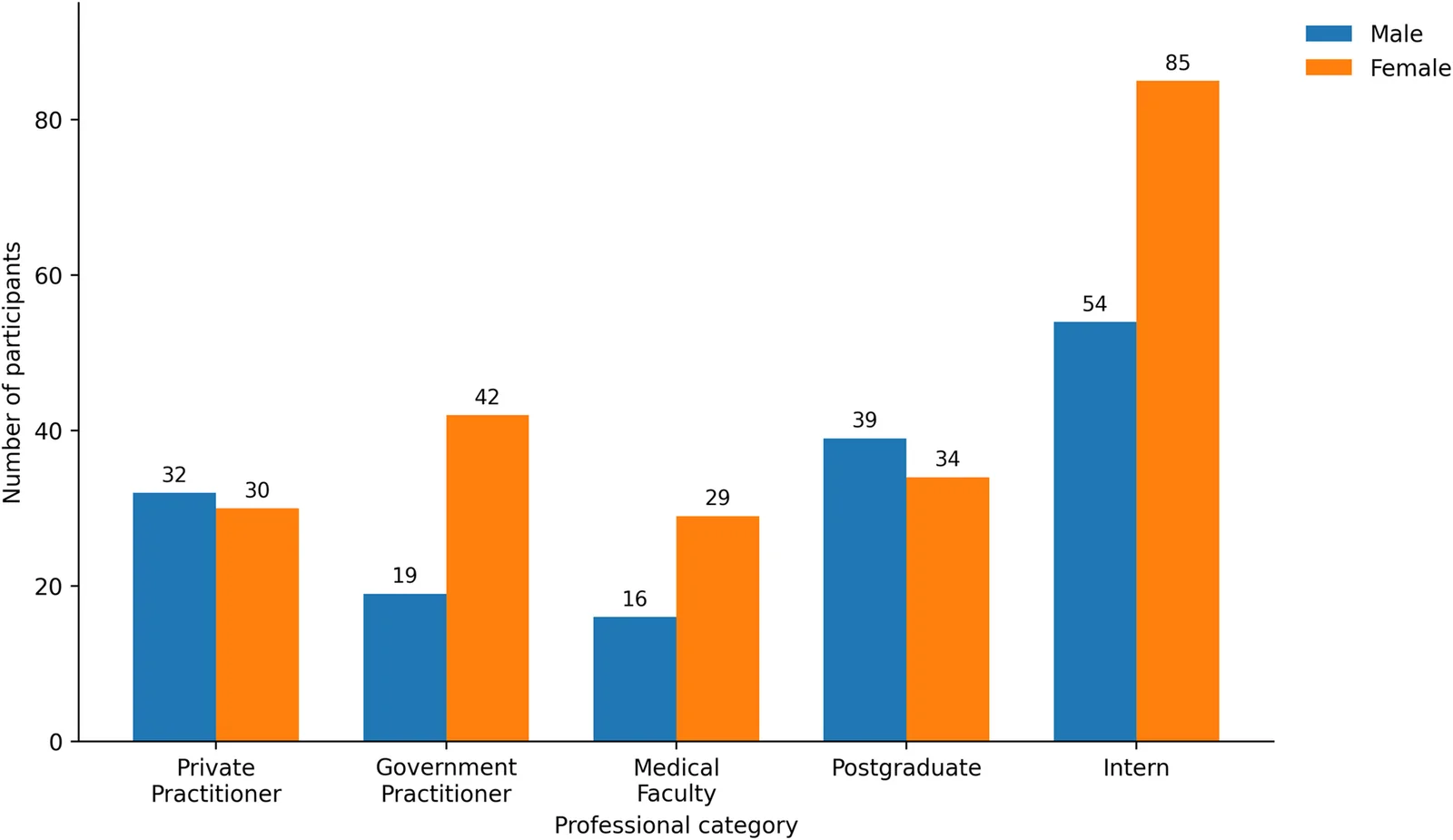
Distribution of participants by professional category and gender.

**Table 1:** Gender-wise distribution of participants by professional category.

Gender	Private Practitioner	Government Practitioner	Medical Faculty	Postgraduate	Intern	Total
Male	32	19	16	39	54	160 (42.1%)
Female	30	42	29	34	85	220 (57.9%)
**Total**	62	61	45	73	139	380 (100%)

**Table 2:** Primary clinical symptoms of periodontal disease by professional category.

Symptom	Private Practitioner	Government Practitioner	Medical Faculty	Postgraduate	Intern	Total (%)
Malodour	9	8	3	6	14	40 (10.5%)
Tooth mobility	18	25	13	11	23	90 (23.7%)
Tooth migration	6	9	16	8	11	50 (13.2%)
Gingival bleeding	12	12	10	30	63	127 (33.4%)
Gingival pain	17	7	3	18	28	73 (19.2%)
**Total**	62	61	45	73	139	380 (100%)

Most participants (90.5%) reported referring patients to dentists during clinical practice; however, only 1.1% based these referrals on knowledge of periodontal–systemic relationships.

Awareness of periodontal disease symptoms was reported by 86.6% of participants. Gingival bleeding was the most frequently identified symptom (33.4%), followed by tooth mobility (23.7%), gingival pain (19.2%), tooth migration (13.2%), and malodour (10.5%). The overall symptom distribution is shown in the redesigned Figure 2.

**Figure 2:**
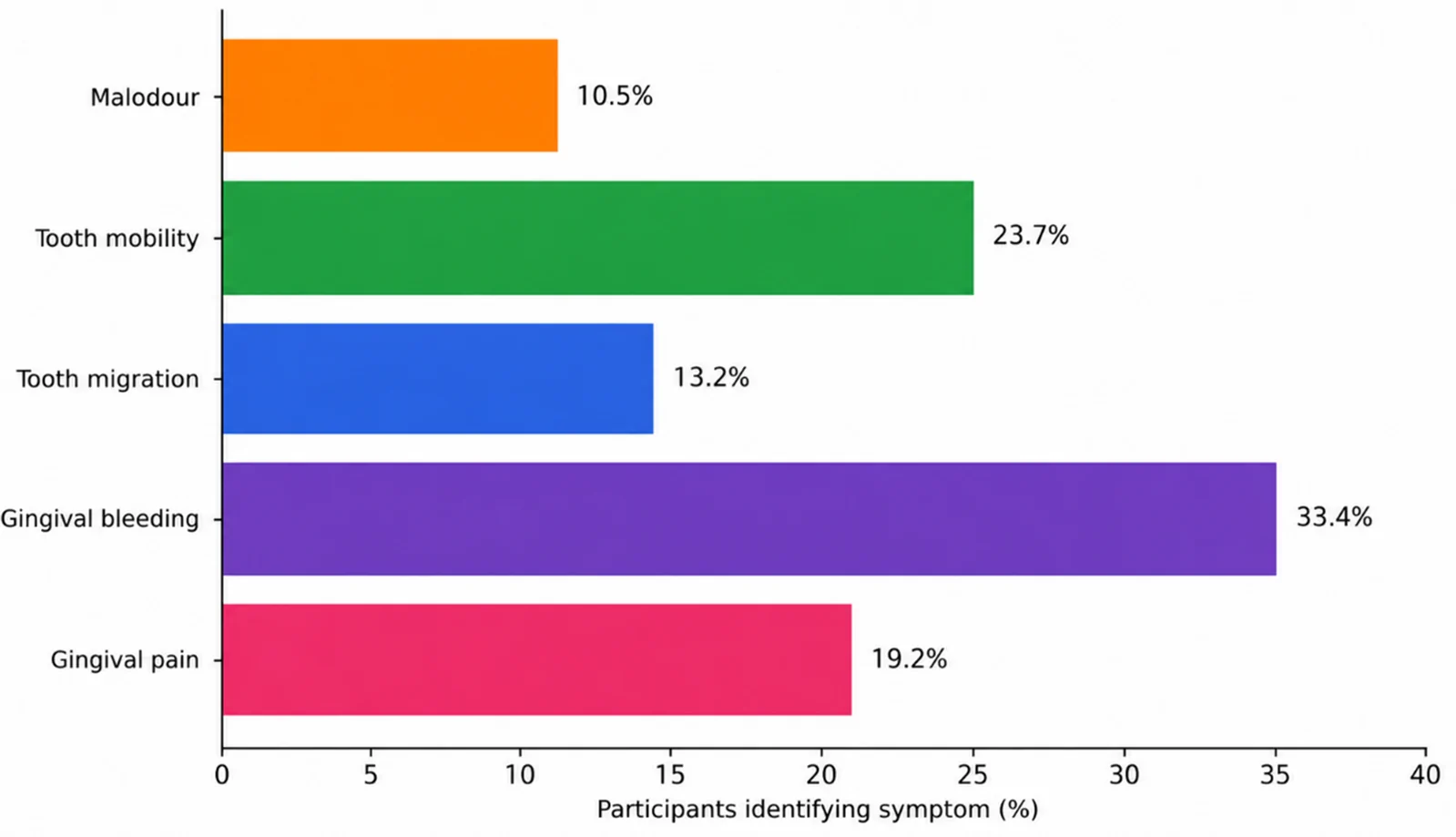
Overall distribution of primary clinical symptoms of periodontal disease identified by participants.

A **chi-square test** demonstrated a statistically significant association between professional category and the primary clinical symptom identified (***p* < 0.001**), indicating variation in symptom recognition across groups.

Mean awareness scores differed significantly among professional groups for diabetes mellitus (*p* = 0.016), adverse pregnancy outcomes (*p* = 0.002), and PCOS (*p* = 0.015). Tukey's *post-hoc* analysis showed significantly higher awareness among government practitioners than interns for diabetes mellitus (*p* = 0.024) and adverse pregnancy outcomes (*p* = 0.002), while medical faculty demonstrated significantly higher awareness than interns for PCOS (*p* = 0.033). Detailed mean ± standard deviation values for all professional groups are presented in Table 3.

**Table 3:** Mean awareness scores for periodontal–systemic associations.

Professional Group	Diabetes (Mean ± SD)	Pregnancy (Mean ± SD)	PCOS (Mean ± SD)
Private Practitioner	3.53 ± 1.03	3.50 ± 1.03	3.56 ± 1.12
Government Practitioner	3.87 ± 0.76	3.75 ± 0.90	3.59 ± 0.78
Medical Faculty	3.76 ± 0.85	3.56 ± 0.78	3.73 ± 0.78
Postgraduate	3.45 ± 1.02	3.32 ± 0.97	3.40 ± 0.98
Intern	3.20 ± 0.95	3.20 ± 0.94	3.25 ± 0.90

## Discussion

The present study provides important insights into the knowledge, awareness, and attitudes of medical professionals regarding the association between periodontal disease and systemic health. Findings of the present study showed considerable variation between how well people know about periodontal disease in general and how well they grasp its systemic effects. Although a high proportion of participants reported referring patients to dentists, only a negligible percentage did so based on knowledge of periodontal–systemic interactions, indicating a critical gap between clinical practice and evidence-based understanding (16, 17).

One of the most notable findings of this study is that referrals to dental professionals were predominantly motivated by the clinical manifestations, such as intraoral lesions, gingival inflammation, and oral malodor, rather than understanding the underlying systemic correlation of periodontal disease (16–18). This approach may delay early intervention in patients with systemic conditions influenced by periodontal inflammation, ultimately compromising patient outcomes (16, 17).

Prior studies supported the association between periodontal disease and systemic conditions (6–11). Chronic periodontal inflammation serves as a main factor that leads to a systemic inflammatory burden through the release of pro-inflammatory cytokines, bacterial endotoxins, and dissemination of periodontal pathogens into the bloodstream (13). These mechanisms are considered to be linked to various factors such as endothelial dysfunction, atherosclerotic plaque formation, and metabolic dysregulation (6, 12, 13). In atherosclerotic plaques, periodontal pathogens like Porphyromonas gingivalis have been identified, which supports the association of periodontal disease with systemic illness. Despite this well-established evidence, the findings of this study indicated that awareness among medical professionals remains suboptimal, particularly among early-career individuals (16, 18).

The bidirectional relationship between periodontal disease and diabetes mellitus remains one of the most extensively studied aspects of periodontal medicine (7). Hyperglycemia damages periodontal tissue and immunological responses, whereas periodontal inflammation also impacts insulin resistance and glycemic management. The present findings indicate that government practitioners showed significantly higher awareness of this relationship compared with interns, suggesting that clinical exposure and experience may play a critical role in enhancing knowledge. However, the relatively lower awareness among interns highlights a deficiency in undergraduate medical education, where oral health is often underemphasized (19).

The lower awareness observed among interns may reflect gaps in undergraduate exposure to oral health and periodontal medicine. Potential curricular gaps include limited structured teaching on periodontal-systemic relationships, limited practical training in basic oral and periodontal examination and referral indications, and few opportunities for interdisciplinary learning with dental professionals. This finding suggests a need for greater integration of oral and periodontal health concepts into undergraduate medical education. In contrast, senior practitioners and faculty may acquire greater awareness through cumulative clinical exposure and continuing professional education. These explanations should be interpreted cautiously because the present survey did not directly measure participants' prior oral-health training, curriculum content, or interdisciplinary exposure. The findings nevertheless support strengthening oral-health content in undergraduate and postgraduate medical curricula and developing joint medical-dental educational activities (16–19).

There was a difference in awareness between periodontal disease and adverse pregnancy outcomes across professional subgroups. Inflammatory mediators generated during periodontal infection may affect the fetal–placental unit and contribute to adverse pregnancy outcomes, including preterm birth and low birth weight (8, 15). The limited awareness in certain groups of medical professionals is an area of concern, as early identification and management of periodontal disease in pregnant women may be helpful in reducing the risk of adverse outcomes.

The study also evaluated awareness regarding the area that gained attention recently, i.e., the association between periodontal disease and polycystic ovary syndrome (PCOS). Recent studies indicated that chronic inflammation and oxidative stress are associated with periodontal disease, which results in metabolic and hormonal imbalances in PCOS (20). Medical faculty had greater awareness compared to interns, reflecting their greater exposure to recent research findings and continuing medical education. However, the overall variability in awareness highlights the need for continuous professional development programs, which may incorporate emerging evidence in periodontal medicine.

Awareness varied across professional groups, with interns showing comparatively lower scores in the domains that differed significantly. This pattern reinforces the need for earlier and more structured exposure to oral-systemic concepts during medical training, while recognizing that the present cross-sectional study cannot determine whether curriculum, clinical experience, continuing education, or other factors caused the observed differences.

These findings align with the results of previous studies conducted globally, which have reported inadequate awareness of oral–systemic relationships among medical professionals (16, 18). The key contributing factor for this inadequate awareness might be lack of integration between medical and dental education. Usually medical curricula focus extensively on systemic diseases, with minimal emphasis on oral health (19). This disparity between disciplines may hinder the development of a holistic approach to patient care.

These findings have a significant impact on both clinical practice and healthcare systems. Improving awareness of periodontal-systemic interaction would help in early diagnosis, promote timely referral, and facilitate interdisciplinary coordination (16, 17). For example, physicians managing patients with diabetes or cardiovascular disease can incorporate oral health assessment into routine clinical evaluation, which can facilitate overall disease management (6, 7, 12). Likewise, collaboration between dentists and obstetricians can play a vital role in improving pregnancy outcomes (8, 15).

From an academic standpoint, the findings emphasize the necessity to integrate oral health into the medical curriculum at both undergraduate and postgraduate levels. Joint clinical training sessions with interdisciplinary teaching modules and collaborative workshops involving both dental and medical students may help bridge the existing knowledge gap. Continuing medical education programs and professional development initiatives should also stress the importance of periodontal-systemic relationships (16–19).

Policy-level changes may be required to promote integrated healthcare delivery in addition to the above-said educational interventions. Incorporating oral health assessments into routine medical check-ups and establishing referral protocols between medical and dental professionals could improve patient outcomes. Public health initiatives aimed at increasing awareness of oral–systemic health among healthcare providers and patients may further enhance preventive care (16, 17).

Future research should focus on evaluating the effectiveness of educational interventions in improving awareness of periodontal–systemic relationships among medical professionals. Studies assessing the impact of interdisciplinary training on referral practices and patient outcomes would be particularly valuable. Furthermore, exploring barriers to collaboration between medical and dental professionals may help identify strategies to enhance integrated care.

### Limitations

Several limitations should be considered when interpreting the findings. Convenience sampling may have introduced selection bias and may limit generalizability. Self-reported questionnaire data may be affected by recall and social-desirability bias, and the cross-sectional design identifies associations but cannot establish temporal or causal relationships. Because recruitment occurred through professional networks, individual contacts, WhatsApp groups, and other group-based electronic platforms, the exact number of individuals who received or viewed the invitation could not be reliably determined; consequently, a conventional response rate could not be calculated. Although the questionnaire demonstrated good internal consistency, further formal psychometric validation in different populations would strengthen the instrument. Prior exposure to oral-health education was not assessed; therefore, the observed differences in awareness cannot be directly attributed to curricular or training differences.

## Conclusion

The present study highlights a significant knowledge gap among medical professionals regarding periodontal–systemic relationships. Although medical professionals commonly referred patients to dentists, these referrals were not consistently guided by awareness of periodontal–systemic associations. Incorporating periodontal–systemic health into undergraduate and postgraduate medical curricula, promoting interdisciplinary continuing education, and establishing structured referral pathways between physicians and dental professionals may improve awareness, strengthen collaborative care, and enhance patient outcomes.

## Data Availability

The original contributions presented in the study are included in the article/Supplementary Material, further inquiries can be directed to the corresponding authors.
