# Supplementary material for: Knowledge, awareness, and attitudes of medical professionals toward the periodontal–systemic disease relationship: a cross-sectional study
Source: Front Oral Health. 2026 Oct 5;7:1931494. doi: 10.3389/froh.2026.1931494 (PMC13678459; doi:10.3389/froh.2026.1931494)
Supplement: Supplementary file 1 [file Supplementaryfile1.docx]

**ANNEXURE**

**QUESTIONNAIRE**

**Part-1**

1. Name:

2. Age

3. Sex: Male/Female/Others

4. Type of work?

Private practitioner

Government practitioner

Medical faculty

Post graduate

Intern

5. The state of practice of the practitioner/pg/intern?

6. Age of the practitioner/pg/intern?

7. Professional experience of practitioner/pg/?

9. What is the type of your institution /workplace?

Public hospital (PHC)	Private hospital	University hospital

**Part-2**

10. Have you ever referred your patient to a dentist?

Yes	No

If your answer is “**Yes**” please answer the next question

11. What was the most frequent reason for referral?

Presence of intra oral lesion

Malodour

Gingival

Oral findings of systemic disease

Knowledge on relationship between periodontal disease and	systemic disease

Before any medical intervention to minimize oral microbial burden

12. Do you know the symptoms of periodontal disease?

Yes	No

13. What is the primary clinical symptom of periodontal disease?

Malodour

Tooth mobility

Tooth migration

Gingival bleeding

Gingival pain

**Part-3**

14. Periodontal disease is a complication of diabetes mellitus

Strongly agree	Agree	Neither agree nor disagree	Disagree	Strongly disagree

15. Diabetic patients should be routinely referred to a dentist

Strongly agree	Agree	Neither agree nor disagree	Disagree	Strongly disagree

16. Active periodontal disease has influence over the glucose level of the diabetic	patient

Strongly agree	Agree	Neither agree nor disagree	Disagree	Strongly disagree

17. Periodontal treatment has effect on the glucose level of the diabetic patient

Strongly agree	Agree	Neither agree nor disagree	Disagree	Strongly disagree

18. HbA1C is the best and most accurate lab test for control of diabetes mellitus patients

Strongly agree	Agree	Neither agree nor disagree	Disagree	Strongly disagree

19. Periodontitis is an independent risk factor to develop myocardial infarction

Strongly agree	Agree	Neither agree nor disagree	Disagree	Strongly disagree

20. Periodontal treatment has effect in reducing systemic inflammatory markers

Strongly agree	Agree	Neither agree nor disagree	Disagree	Strongly disagree

21. Gingival enlargement is a common oral manifestation of Nifedipine

Strongly agree	Agree	Neither agree nor disagree	Disagree	Strongly disagree

22. Dental treatment is proceeded without stopping aspirin

Strongly agree	Agree	Neither agree nor disagree	Disagree	Strongly disagree

23. Aspire need to be stopped >3days before proceeding dental treatment

Strongly agree	Agree	Neither agree nor disagree	Disagree	Strongly disagree

24. Active periodontal disease leads to preterm delivery and low birth weight

Strongly agree	Agree	Neither agree nor disagree	Disagree	Strongly disagree

25. Second trimester of pregnancy is the best period to perform elective dental treatment

Strongly agree	Agree	Neither agree nor disagree	Disagree	Strongly disagree

26. Presence of PCOS increase the risk of periodontitis

Strongly agree	Agree	Neither agree nor disagree	Disagree	Strongly disagree

27. Periodntities is associated with increased covid19 complications

Strongly agree	Agree	Neither agree nor disagree	Disagree	Strongly disagree
